# 肺鳞癌驱动基因研究进展

**DOI:** 10.3779/j.issn.1009-3419.2014.05.13

**Published:** 2014-05-20

**Authors:** 卫 洪, 沂平 张

**Affiliations:** 310022 杭州，浙江省肿瘤医院，浙江省胸部肿瘤诊治技术研究重点实验室 Zhejiang Cancer Hospital, Zhejiang Key Laboratory of the Diagnosis & Treatment Technology on Thoracic Oncology, Hangzhou 310022, China

**Keywords:** 肺肿瘤, 鳞癌, 癌基因, Lung neoplasms, Squamous cell carcinoma, Oncogenes

## Abstract

肺癌在癌症相关死亡中是居于首位的恶性肿瘤。鳞癌是仅次于腺癌的非小细胞肺癌（non-small cell lung cancer, NSCLC）最常见的组织学类型。一些负责恶性肿瘤的发生和维持相关分子改变被称为驱动基因。近来研究证实肺鳞癌也具有与致癌作用及靶向药物疗效有关的独特分子特征。目前发现约40%肺鳞癌已经找到驱动基因，其中纤维母细胞生长因子受体1（fibroblast growth factor receptor 1, FGFR1）起重要作用。本文将对肺鳞癌驱动基因进行综述。

## Abstract (en)

**Background and objective** Lung cancer is the leading cause of cancer-related deaths worldwide. Next to adenocarcinoma, squamous cell carcinoma (SCC) of the lung is the most frequent histologic subtype in non-small cell lung cancer. Several molecular alterations have been defined as " driver oncogenes" responsible for both the initiation and maintenance of the malignancy. The squamous cell carcinoma of the lung has recently shown peculiar molecular characteristics which relate with both carcinogenesis and response to targeted drugs. So far, about 40% of lung squamous cell carcinoma has been found harbouring driver oncogenes, in which fibroblast growth factor receptor 1 (FGFR1) plays important roles. In this review, we will report the mainly advances on some latest driver mutations of squamous cell lung cancer.

癌基因成瘾（oncogene addiction）是指某些肿瘤维持其恶性生物学表型依赖于某个或某些活化癌基因的现象，这些癌基因也称为驱动癌基因（driver oncogenes）^[1]^。癌细胞需要驱动癌基因持续发挥功能，而正常细胞则不需要。因此，以癌基因为治疗靶点，可以使靶向药物特异性地杀伤肿瘤细胞，而不损伤正常细胞。

肺癌居全球范围内癌症死亡原因的首位，非小细胞肺癌（non-small cell lung cancer, NSCLC）约占肺癌的80%，鳞癌是仅次于腺癌的NSCLC最常见的组织学类型，导致全世界每年约有40万患者死亡^[2]^。2004年世界卫生组织（World Health Organization, WHO）的分类中，将鳞癌分为乳头状、基底样、透明细胞及小细胞4种亚型。一般认为，肺鳞癌均表达癌基因*p63*而不表达甲状腺转录因子-1（thyroid transcription factor-1, TTF-1）。多项研究^[3-5]^认为，P40（DeltaNp63），P63的同型异构体抗体，对诊断肺鳞癌特异性更高。

以驱动基因为靶点的肺腺癌的治疗新进展使得人们对靶向治疗充满憧憬，例如多个表皮生长因子受体酪氨酸激酶活性抑制剂（epidermal growth factor receptor tyrosine kinase inhibitor, EGFR-TKI）在治疗*EGFR*基因突变的NSCLC^[6-9]^以及克唑替尼（crizotinib）治疗棘皮动物微管相关蛋白样4-间变淋巴瘤激酶融合基因阳性（echinodern microtubule-associated protein-like 4 anaplastic lymphoma kinase, EML4-ALK）的晚期NSCLC，均取得突出疗效，有效率可以高达60%-80%^[10]^。尽管肺鳞癌的研究进展较慢，但近年仍取得了一些进展^[2]^，本文对肺鳞癌驱动基因的研究进展作一综述。

## 1 驱动基因筛查研究

近年来，系统地进行肺鳞癌驱动基因的研究比较少。一项来自癌症基因组图谱研究网络（Cancer Genome Atlas Research Network, CGARN）的研究，对包括178例初治肺鳞癌进行表观基因组学和基因组学研究，已经发表的包括18个基因的结果表明，发现平均每个标本有360个外显子突变，165个基因重排，323个基因拷贝数异常。抑癌基因*TP53*突变几乎见于所有的标本，并存在多种的信号通路异常，包括泛素连接酶复合体衔接蛋白1（Kelch-like ECH-associated protein 1, KEAP1）/小鼠核因子E2相关蛋白2（Rat nuclear factorerythroid 2-related factor 2, NFE2L2）占所有标本的34%，鳞癌分化基因占44%，磷脂酰肌醇3-激酶/蛋白激酶B/哺乳动物雷帕霉素靶蛋白（phosphatidylinositol-3-kinase/protein kinase B/mammalian target of Rapamycin, PI3K/Akt/mTOR）信号通路占47%等^[2]^。而更早期的一项磷酸酪氨酸信号通路的研究^[11]^中，包括41个NSCLC细胞株和多达150个标本，揭示多种激酶通路的基因组学异常，包括盘状结构域受体（discoidin domain receptor tyrosine kinase, DDR）1和2，血管内皮生长因子受体（vascular endothelial growth factor receptor, VEGFR）1和2，血小板源性生长因子受体α多肽（platelet-derived growth factor receptor alpha, PDGFRα）等。这些异常的发现使得靶向肺鳞癌的治疗成为可能。

## 2 肺鳞癌驱动基因

尽管已经筛查发现许多肺鳞癌的差异表达基因，但目前比较公认的并已经进入临床研究驱动基因并不多，简述如下。

### 2.1 FGFR1

纤维母细胞生长因子受体1（fibroblast growth factor receptor 1, FGFR1）是FGFR家族成员（包括FGFR1、FGFR2、FGFR3、FGFR4）之一，是一种酪氨酸跨膜激酶受体，编码基因位于8p12^[12]^，在正常生理功能中有重要作用，参与胚胎的发育、细胞增殖、分化和血管生成。它通过4条通路来调节，即MAPK、PI3K-AKT、STAT及磷脂酶Cγ^[13]^。通过荧光原位杂交（fluorescence *in situ* hybridization, FISH）方法，Weiss及其研究小组^[14]^首次报道：在155例肺鳞癌标本中22%例标本存在FGFR1扩增，而581例非鳞癌患者中，仅1%存在*FGFR1*基因扩增。结果提示，FGFR1扩增可能是肺鳞癌特有的分子标志。进一步动物试验中发现在裸鼠致瘤模型中运用FGFR1抑制剂PD173074可使存在*FGFR1*基因扩增的肺鳞癌小鼠的肿瘤明显缩小，肺癌细胞的生长依赖于FGFR1的扩增。其他研究小组的研究^[14, 15]^也发现，肺鳞癌FGFR1的扩增的比例约20%，而腺癌则小于5%。因此，目前研究认为*FGFR1*是肺鳞癌驱动基因。

有研究^[16]^检测262例肺鳞癌标本，发现肺鳞癌FGFR1扩增与吸烟相关，正吸烟、曾吸烟及从不吸烟者，扩增比例分别为28.9%、2.5%和0，这需要进一步观察验证。韩国的这项研究还发现，在手术切除的标本中检测到FGFR1扩增的患者预后明显（无疾病生存时间26.9个月*vs* 94.6个月，总生存时间51.2个月*vs* 115个月）^[14]^。也有研究在高加索人群226例患者的中发现FGFR1扩增与预后无关^[17]^，推测得出不同的结论可能与不同的扩增定义设定或人种差异等相关，需要进一步观察。

目前有多项针对FGFR1的临床研究正进行中，dovitinib作用于FGFR1扩增的肺鳞癌的Ⅱ期临床研究正进行中（NCT01861197）；AZD4547在选择性的人群中正针对FGFR1和FGFR2的扩增（NCT01795768）进行早期临床研究；BGJ398针对FGFR1和FGFR2的扩增及FGFR突变的人群也正进行中（NCT01004224）。

### 2.2 PI3KCA PI3K/Akt信号传导通路

PI3KCA PI3K/Akt信号传导通路与肿瘤细胞的存活、代谢、运动及血管生成相关。*PI3KCA*基因突变编码PI3K的催化亚单位p110α，其突变集中于外显子9和20，在鳞癌较腺癌常见，报道的突变率在2%-6.5%^[18, 19]^。

肺鳞癌*PI3KCA*基因突变与*EGFR*或*KRAS*突变可能并不互相排斥^[19, 20]^。一项包含1, 125例患者的术后病理组织一项研究发现，突变患者占2%（23例），16例（70%）合并其它突变^[21]^。但也有学者认为肺鳞癌有时候与腺鳞癌及分化差的腺癌难以区别，他们以DeltaNp63 (p40)(+)/TTF-1(-)作为肺鳞癌的免疫组化诊断标准，发现*PI3KCA*基因突变比例约4%（4/95），没有发现合并*EGFR*或*KRAS*突变^[22]^。

*PI3KCA*基因的扩增远较突变常见，鳞癌可以高达33.1%，而腺癌（6.2%）和小细胞癌（4.7%）^[19]^，体外研究^[23]^鳞癌细胞株发现*PI3KCA*基因扩增促进肿瘤细胞生长。目前认为，*PI3KCA*基因突变与扩增的关系并未进行充分的研究，一般认为*PI3KCA*基因突变，对肿瘤生物学行为影响更大，目前针对*PI3KCA*基因突变的单药Ⅱ期研究，包含肺癌、乳腺癌、结直肠癌等多种癌症，如BKM120（NCT01501604）已经结束入组。

口服PI3K激酶抑制剂-GDC-0941，针对的是非选择人群的研究，有两项研究，分别是与化疗联用的治疗肺癌的加或不加贝伐珠单抗的（NCT00974584研究）及XL147治疗多种实体瘤的NCT00756847研究，他们也已经结束入组。

### 2.3 DDR2盘状结构域受体（discoidin domain receptor 2, DDR2）

DDR2的编码基因位于1q23.3，是调节肿瘤细胞粘附、增殖和迁移的受体酪氨酸激酶^[12]^。它可以被Ⅰ型胶原激活，与Src及Shc相互作用^[24]^。*DDR2*突变后，可以改变激酶活性，改变与配体的结合及改变DDR2的位置^[25]^。Hammermann等^[26]^筛查290例肺鳞癌标本，发现*DDR2*基因突变频率约为3.8%。体外研究发现，应用RNA干扰的技术敲除*DDR2*基因，或多靶点激酶抑制剂达沙替尼（dasatinib），可以选择性杀死*DDR2*突变的肺鳞癌细胞。

肺鳞癌*DDR2*突变率虽然不高，一项针对DDR2突变肺鳞癌的临床研究已经结束入组（NCT 01491633），若研究发现肺鳞癌*DDR2*突变者对达沙替尼确实有效，将会对该亚型患者治疗带来突破性的研究进展。

## 3 其他潜在的候选驱动基因

另有一些基因突变率更低，但有多项药物已经进行中，对具有这些分子特征的肺鳞癌患者的靶向治疗带来新的希望。

### 3.1 *MET*基因扩增

*MET*基因是一种编码HGFR蛋白原癌基因，是一种酪氨酸激酶膜受体，位于染色体7q21-q3，其与配体肝细胞生长因子（hepatocyte grow factor, HGF）均促进肿瘤发生^[27]^。MET扩增可见于鳞癌和腺癌。MET扩增与EGFR-TKI继发耐药有关，有报道^[28]^认为是通过激活ERBB-3通路相关。尽管有报告^[29]^认为在肺癌的扩增比例约3%-21%，但由于这是一种低水平的扩增，有学者认为突变的比率要低的多，估计肺鳞癌为1%，腺癌为2%。

多项MET抑制剂正研究中，目前的研究一般均针对MET过表达的NSCLC，并非仅针对鳞癌。例如：克唑替尼是MET和ALK的双重抑制剂；多靶点药物cabozantinib（XL184）以MET、RET和VEGF2为靶点^[30]^；Tivantinib（ARQ197）则是以MET为靶点的小分子药物，但有一定的细胞毒性^[31]^；而单克隆抗体MetMAb联合厄洛替尼治疗复治NSCLC^[32]^。

### 3.2 BRAF

BRAF是KRAS下游的丝氨酸/苏氨酸激酶，将RAS鸟苷三磷酸连接到丝裂原活化蛋白激酶家族的下游蛋白，控制细胞增殖。RAF激酶家族包括3个成员：ARAF、BRAF和RAF-1（也称为CRAF）^[33]^。*BRAF*突变与酶活性增加相关，导致MAPK2和MAPK3组成型活化。*BRAF*突变最初是在黑色素瘤中被发现，约80%的突变，影响激酶结构域中外显子15的Val600残基。一般认为其突变在腺癌和鳞癌相当，约2%左右，但在肺癌的突变中与在恶性黑色素瘤中的常见突变V600E不同，值得进一步研究^[33]^。现已研发出多个BRAF抑制剂（PLX4032、XL281、selumetanib、GSK2118436），其中GSK2118436治疗*BRAF*突变肺癌（包括鳞癌）的研究正进行中（NCT01336634）。尽管BRAF小分子抑制剂PLX4032（vemurafenib）对*BRAF* V600E突变阳性的黑色素瘤有非常明显的疗效^[34]^，其对肺癌的疗效还有待评价。

## 4 展望

目前EGFR-TKI的出现使得肺癌的靶向治疗成为现实，但针对肺鳞癌的靶向治疗研究进展相比腺癌明显比较慢，到目前为止尚未开展一项Ⅲ期临床研究。目前也仅发现约40%肺鳞癌患者携带不同的驱动基因，还有相当多的驱动基因未被发现。肿瘤的形成是一个多基因参与的异常复杂的过程，现有的靶向药物如小分子激酶抑制剂大多是针对一个靶点而设计，因此只有发现更多的驱动基因，阐明基因之间的相互作用关系，开发出更多的靶向药物，联合应用或联合其它的治疗方式，才有可能收到理想的疗效。

总之，真正实现肺鳞癌的个体化靶向治疗仍然任重而道远。
